# Supplementary material for: SMAD7 Sustains XIAP Expression and Migration of Colorectal Carcinoma Cells
Source: Cancers (Basel). 2024 Jun 28;16(13):2370. doi: 10.3390/cancers16132370 (PMC11240366; doi:10.3390/cancers16132370)
Supplement: Supplementary file 1 [file cancers-16-02370-s001.zip › cancers-3050378-supplementary.docx]

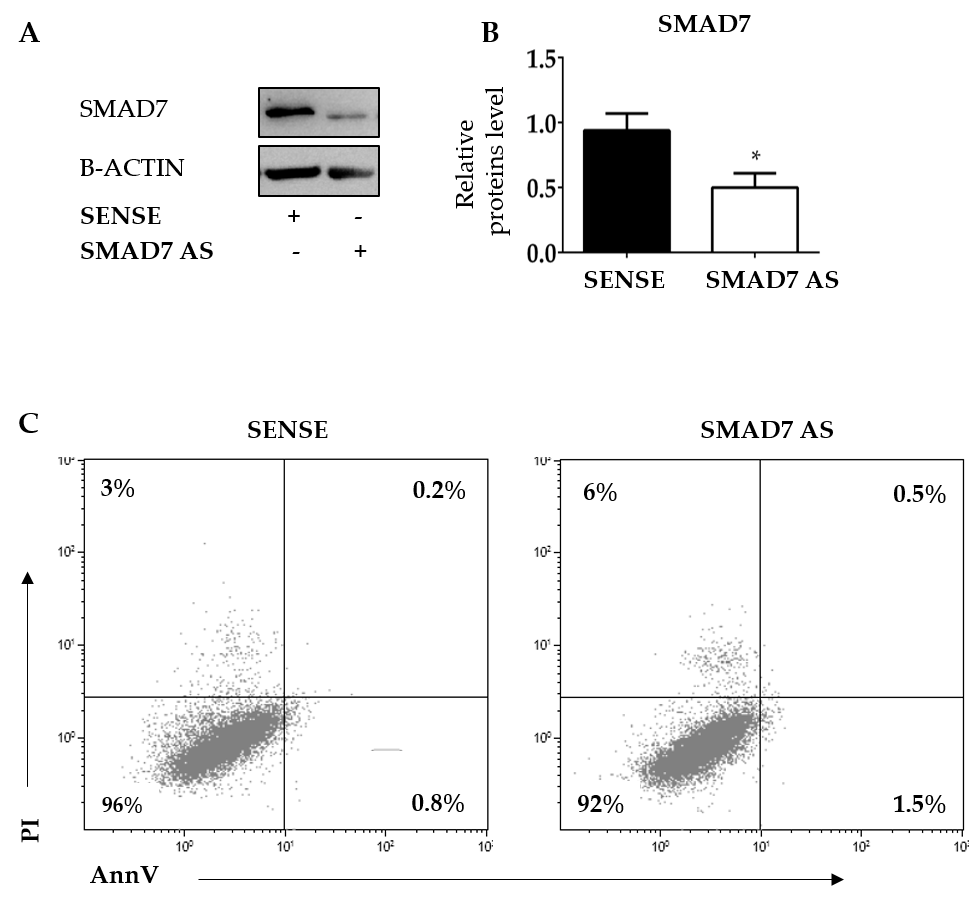


Figure S1. (A) Representative Western blot showing down-regulation of SMAD7 in HCT116 cells transfected with SMAD7 antisense oligonucleotide (AS). Cells were transfected with SMAD7 sense or AS for 24 hours and then total extracts were measured for SMAD7 and β-actin by Western blotting. Panel (B) shows the quantitative analysis of SMAD7 and B-ACTIN, as evaluated by the densitometry scanning of Western blots. Values indicate the percentage (%) of three independent experiments. Differences were analyzed using a two-tailed Student’s *t*-test (* *p* < 0.05). (C) HCT116 cells were transfected as above and the percentages of Annexin V (AV) and/or propidium iodide (Pi) were evaluated by flow cytometry. A representative dot-plot is shown.


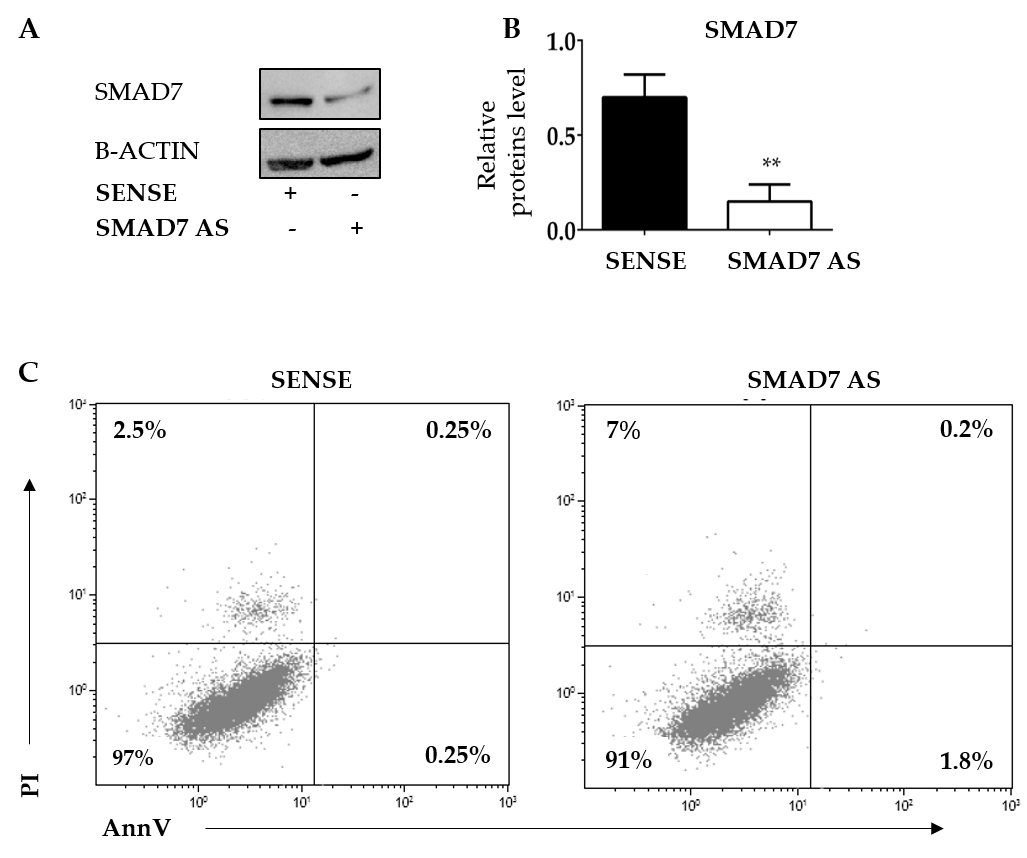


Figure S2. (A) Representative Western blot showing down-regulation of SMAD7 in DLD1 cells transfected with SMAD7 antisense oligonucleotide (AS). Cells were transfected with SMAD7 sense or AS for 24 hours and then total extracts were measured for SMAD7 and β-actin by Western blotting. Panel (B) shows the quantitative analysis of SMAD7 and B-ACTIN, as evaluated by the densitometry scanning of Western blots. Values indicate the percentage (%) of three independent experiments. Differences were analyzed using a two-tailed Student’s *t*-test (** *p* < 0.01). (C) DLD1 cells were transfected as above and the percentages of Annexin V (AV) and/or propidium iodide (Pi) were evaluated by flow cytometry. A representative dot-plot is shown.


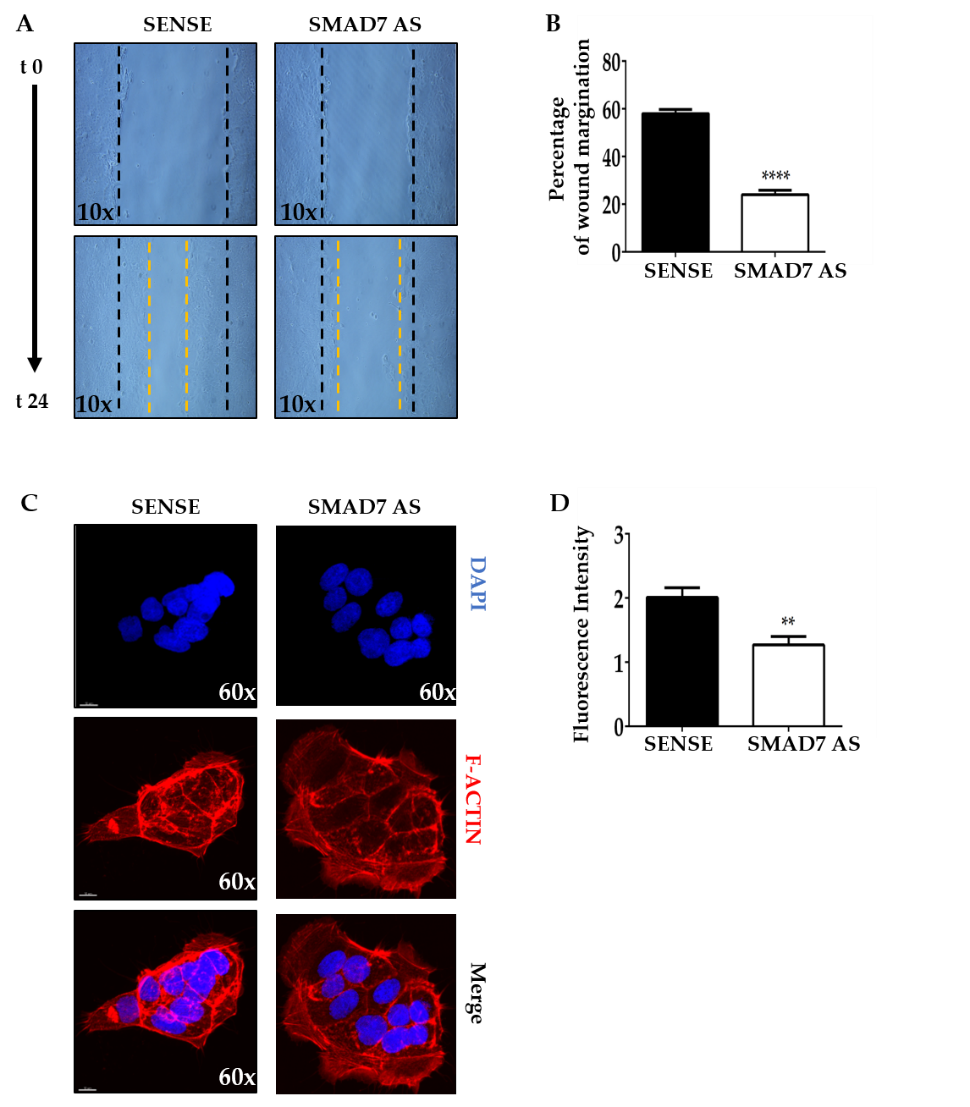


Figure S3. Smad7 knockdown in DLD1 cells reduces the migration rate and the formation of F-ACTIN filaments. (A) DLD1 cells were transfected with either SMAD7 sense or AS for 24h. Representative images of cell migration captured at time 0 and 24h by a phase-contrast microscope (10X). The figure is representative of three separate experiments in which similar results were obtained. (B) Quantitative analysis shows the percentage of wound margination at 24 h in comparison to that measured at time 0. The values indicate the mean ± SD; the differences were analyzed using a two-tailed Student’s *t*-test (**** *p* < 0.0001). (C) DLD1 cells were transfected as above. Representative confocal microscopy images showing F-ACTIN (red) and DAPI (blue) staining (60X). The figure is representative of three separate experiments in which similar results were obtained. (D) Quantitative analysis of the fluorescence intensity in cells cultured as above. The values indicate the mean ± SD; the differences were analyzed using a two-tailed Student’s *t*-test (** *p* < 0.01).


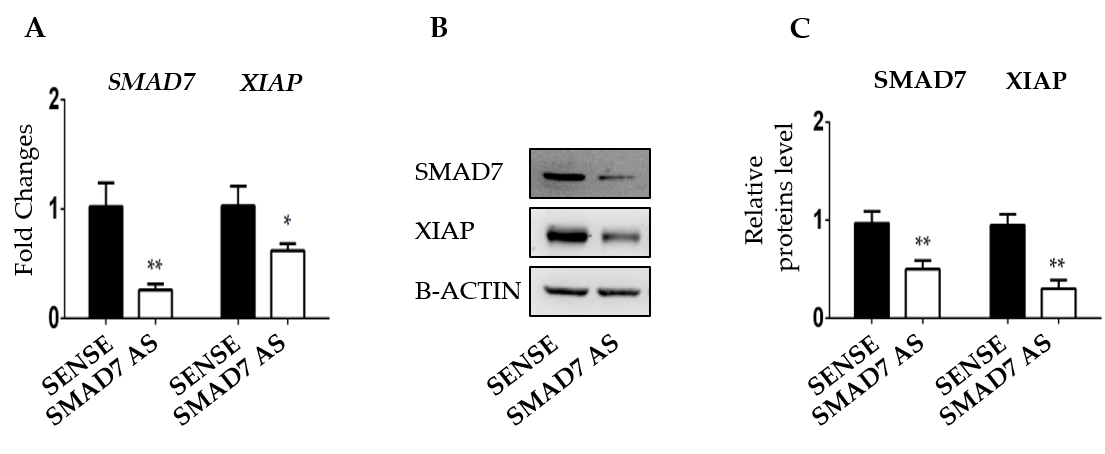


Figure S4. DLD1 cells transfected with either SMAD7 sense or AS for 24h. A. SMAD7 and XIAP mRNA transcripts were evaluated by real-time polymerase chain reaction. Levels were normalized to B2m. Values show the mean ± SD of three independent experiments. Differences were analyzed using a two-tailed Student’s *t*-test (* *p* < 0.05, ** *p* < 0.01). (B) Cells were transfected, as indicated in A, and the protein content of SMAD7 and XIAP were analyzed by Western blotting. Panel (C) shows the quantitative analysis of SMAD7, XIAP, and B-ACTIN, as evaluated by the densitometry scanning of Western blots. Values indicate the mean ± SD of three independent experiments; Differences were analyzed using a two-tailed Student’s *t*-test (** *p* < 0.01).


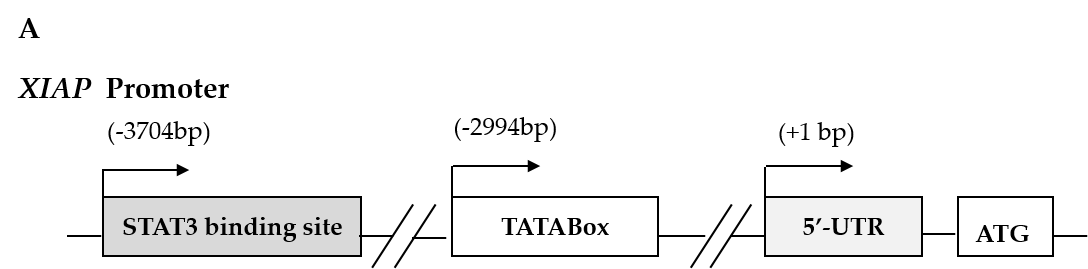


Figure S5. Schematic representation of the human XIAP promoter (5000 bp upstream of the XIAP gene, ENSEMBL, transcript ID ENST00000371199.8) as found with bioinformatics analysis conducted with JASPAR. The sentence “STAT3 binding site” refers to the “MA0144.3 sequences from the JASPAR tool.


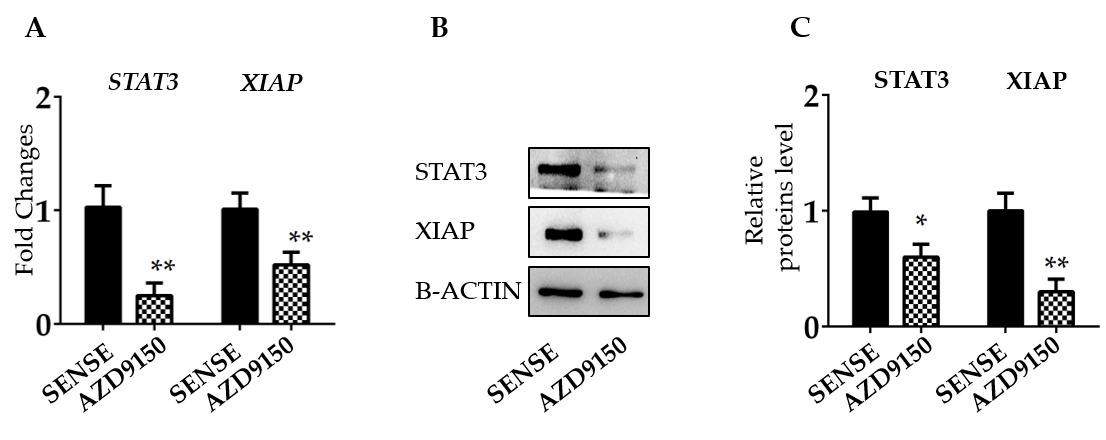


Figure S6. DLD1 cells were transfected with either STAT3 sense or AS (AZD9150) for 24h and STAT3 and XIAP mRNA transcripts were evaluated by real-time polymerase chain reaction. Levels were normalized to B2m. Values show the mean ± SD of three independent experiments. Differences were analyzed using a two-tailed Student’s *t*-test (** *p* < 0.01). (B) Cells were transfected, as indicated in A, and STAT3 and XIAP proteins were analyzed by Western blotting. Panel (C) shows the quantitative analysis of STAT3, XIAP, and B-ACTIN, as evaluated by the densitometry scanning of Western blots. Values indicate the mean ± SD of three independent experiments; Differences were analyzed using a two-tailed Student’s *t*-test (* *p* < 0.05, ** *p* < 0.01).
