# Supplementary figures and images for: SMAD7 Sustains XIAP Expression and Migration of Colorectal Carcinoma Cells
Source: Cancers (Basel). 2024 Jun 28;16(13):2370. doi: 10.3390/cancers16132370 (PMC11240366; doi:10.3390/cancers16132370)

File S1. Uncropped Western blots.

Figure 2

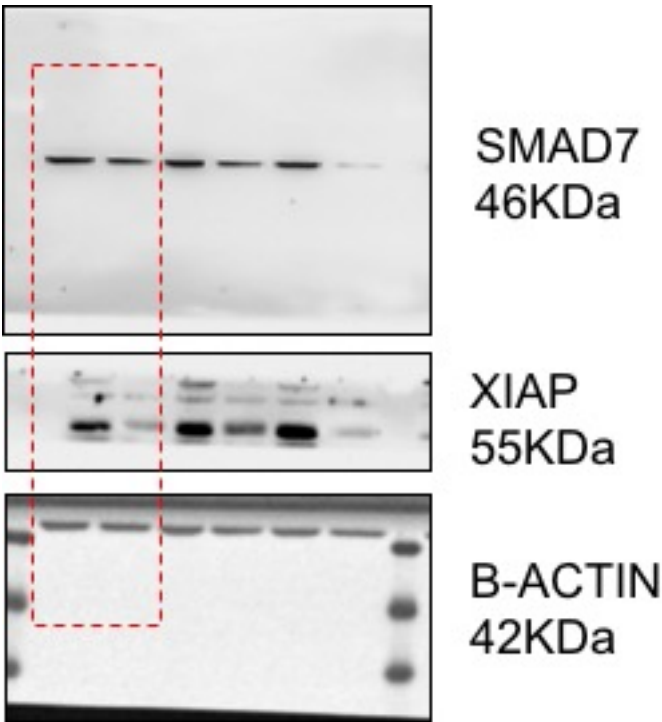

Figure 3

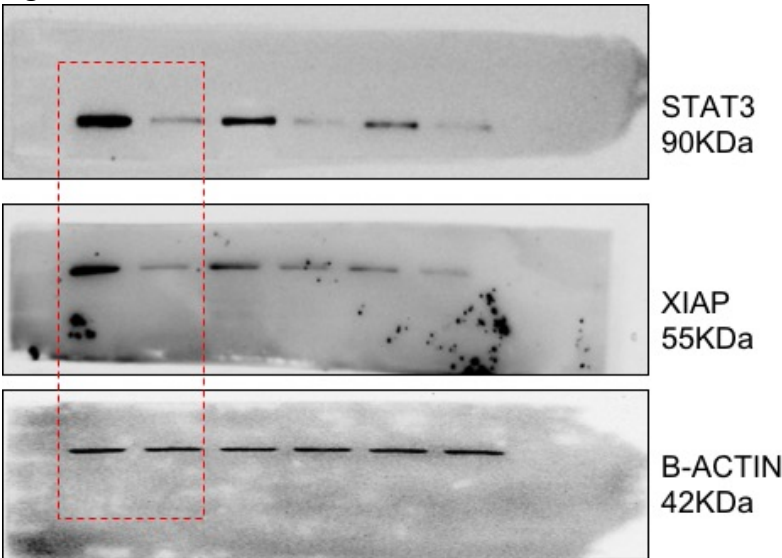

Figures S1A and S2A

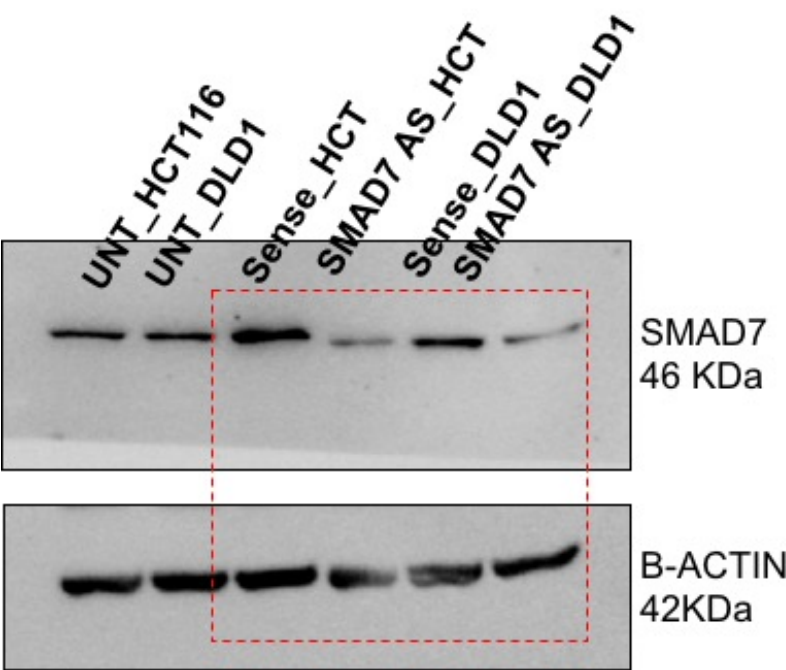

Figure S4

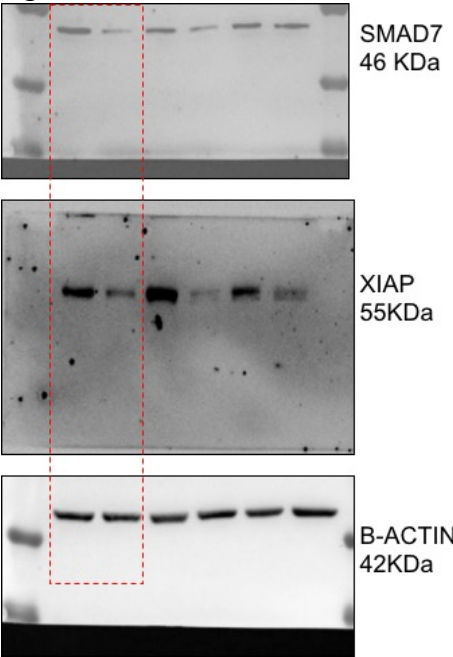

Figure S6

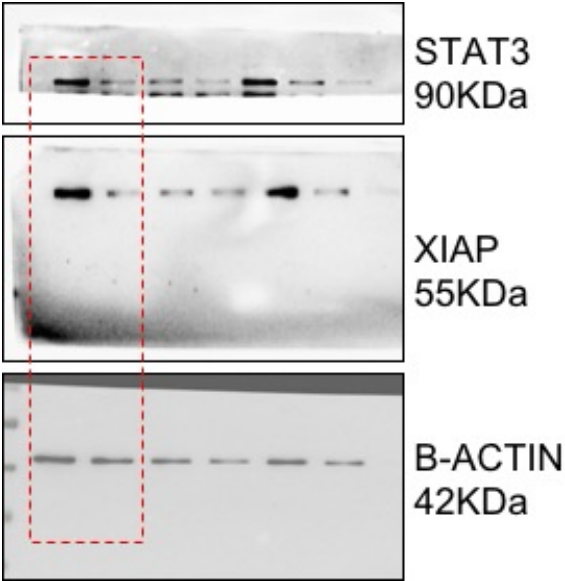

Supplement: Supplementary file 1 [file cancers-16-02370-s001.zip › cancers-3050378-uncropped western blots.pdf]
